# A systematic review of digital technology to evaluate motor function and disease progression in motor neuron disease

**DOI:** 10.1007/s00415-022-11312-7

**Published:** 2022-08-09

**Authors:** Emily Beswick, Thomas Fawcett, Zack Hassan, Deborah Forbes, Rachel Dakin, Judith Newton, Sharon Abrahams, Alan Carson, Siddharthan Chandran, David Perry, Suvankar Pal

**Affiliations:** 1 Centre for Clinical Brain Sciences, The University of Edinburgh, Edinburgh, Scotland, UK; 2 Anne Rowling Regenerative Neurology Clinic, The University of Edinburgh, 49 Little France Crescent, Edinburgh, EH16 4SB Scotland, UK; 3 Euan MacDonald Centre for MND Research, The University of Edinburgh, Edinburgh, Scotland, UK; 4 The School of Medicine and Veterinary Medicine, The University of Edinburgh, Edinburgh, Scotland, UK; 5 Human Cognitive Neurosciences, Psychology, School of Philosophy, Psychology and Language Sciences, The University of Edinburgh, Edinburgh, Scotland, UK; 6 UK Dementia Research Institute, The University of Edinburgh, Edinburgh, Scotland, UK

**Keywords:** Amyotrophic lateral sclerosis, Motor neuron disease, Devices, Sensors, Clinical trials

## Abstract

Amyotrophic lateral sclerosis (ALS) is the most common subtype of motor neuron disease (MND). The current gold-standard measure of progression is the ALS Functional Rating Scale—Revised (ALS-FRS(R)), a clinician-administered questionnaire providing a composite score on physical functioning. Technology offers a potential alternative for assessing motor progression in both a clinical and research capacity that is more sensitive to detecting smaller changes in function. We reviewed studies evaluating the utility and suitability of these devices to evaluate motor function and disease progression in people with MND (pwMND). We systematically searched Google Scholar, PubMed and EMBASE applying no language or date restrictions. We extracted information on devices used and additional assessments undertaken. Twenty studies, involving 1275 (median 28 and ranging 6–584) pwMND, were included. Sensor type included accelerometers (*n* = 9), activity monitors (*n* = 4), smartphone apps (*n* = 4), gait (*n* = 3), kinetic sensors (*n* = 3), electrical impedance myography (*n* = 1) and dynamometers (*n* = 2). Seventeen (85%) of studies used the ALS-FRS(R) to evaluate concurrent validity. Participant feedback on device utility was generally positive, where evaluated in 25% of studies. All studies showed initial feasibility, warranting larger longitudinal studies to compare device sensitivity and validity beyond ALS-FRS(R). Risk of bias in the included studies was high, with a large amount of information to determine study quality unclear. Measurement of motor pathology and progression using technology is an emerging, and promising, area of MND research. Further well-powered longitudinal validation studies are needed.

**Supplementary Information:**

The online version contains supplementary material available at 10.1007/s00415-022-11312-7.

## Introduction

Amyotrophic lateral sclerosis (ALS) is the most common subtype of motor neuron disease (MND), a neurodegenerative condition characterised by progressive loss of motor function. Only 51.3% of people with MND (pwMND) survive more than 12 months from diagnosis [1]. The only globally licensed disease-modifying treatment for MND, riluzole, has limited efficacy and extends survival by just 2–3 months [2]. There is an urgent clinical need for more effective therapies and many clinical trials are in progress or planned [3]. Accurate measurement of symptom progression in MND is a significant challenge in both clinical and research settings.

The current gold standard for evaluating physical symptom severity and disease progression is the Amyotrophic Lateral Sclerosis Functional Rating Scale—Revised (ALS-FRS(R)), a questionnaire-based assessment, evaluating the presence and resulting disability, of physical symptoms commonly affecting people with MND. However, the ALS-FRS(R) is reliant on clinical judgement, subjective reporting and pwMND’s recollection of symptoms [4]. A sensitive measure of disease trajectory is an essential requirement of clinical trial outcome measures. Instruments such as the ALS-FRS(R) generate composite scores that may not be sensitive to smaller changes in function, necessitating large trial sample sizes, more frequent assessments points and longer duration follow-up, increasing participant burden [5].

Remote monitoring of function may also improve clinical care delivery [6]. Collecting information between appointments may facilitate delivery of more personalised care [6, 7]. The prognostic capacity of devices is an area of active investigation in a range of neurological conditions [8]. Improved ability to predict disease trajectory and early identification of impairment may also help with care planning.

MND is characterised by clinical heterogeneity in site of onset and disease progression [9]. The different types of devices available offer the opportunity to evaluate different body regions, whilst enabling each person to act as their own baseline for detecting change and progression [10].

The potential of greater sensitivity for detecting change with these devices and their implementation as alternative outcome measures may lead to significant reduction in sample size requirements for trials by 30.3% and 44.6% for 18-months trials [10]. Smaller sample size requirements for detecting effects of new medicines reduces trial delivery costs and shortens timelines for trials [10].

Using devices for remote data collection also reduces the need for frequent trial appointments for participants. Decentralised trial delivery offers the opportunity for more frequent assessments, potentially further reducing sample size requirements, for example weekly versus monthly ALS-FRS(R) completion [11, 12]. The opportunity for remote data collection also offers trialists the opportunity to reduce the burden of trial participation on people with MND and optimise retention [13].

Activity monitors evaluate changes in participants’ overall capability for engaging in physical activity [11], whilst wearable devices containing an inertial measurement unit (IMU) enable researchers to provide a picture of an individual’s ability to move their limbs [10]. An IMU is contained within a wearable device and used to measure velocity, orientation and gravitational force, which in turn can provide detailed information on the participant’s movements. Within the IMU are an accelerometer, gyroscope and magnetometer sensor. Accelerometers measure acceleration from inertia (movement from a resting baseline), gyroscope measure angular rotation (direction of movement) with the magnetometer improving the accuracy of the gyroscope’s determination of direction [14].

Smartphones are also used to collate passive (automatically collected by the phone itself) and active (entered by participants into specialised apps or web forms) data on function, symptoms and daily activity [13]. Electrical impedance myography devices apply a low-intensity electrical current to a limb, to evaluate an area of muscle and through repeated measurements, we can evaluate changes in the structure and composition of the muscle as it degrades due to disease progression, offering a potential alternative biomarker for people with MND [15]. Dynamometry is also focussed on the muscles, evaluating decline through measuring strength of pressure muscles are capable of, this can be focussed on a specific area of the body (eg hand function and grip strength) or more global decline [16].

In other progressive motor disorders, such as Parkinson’s disease, devices have offered an alternative method of continuous and objective monitoring of motor symptoms in both clinical care and trial delivery [17]. The clinical utility of these devices and their potential suitability as trial outcome measures, in people with MND has steadily gained attention in research. In this study, we will explore the current landscape of research in this area: the devices used, aspects of MND evaluated and directions for future work.

### Aim

The aims of this study are to improve understanding of the types of device currently used in research to evaluate motor symptoms in pwMND, and to explore if studies consider the acceptability of devices to participants, and the feasibility their use for routine data collection in MND.

### Objectives

The objective of this study is to systematically review previous studies reporting use of devices in people with MND and their suitability to evaluate motor progression in research and clinical care for pwMND.

### Hypothesis

We hypothesise that the majority of studies in this area will be exploratory: utilising small sample sizes and shorter lengths of follow-up but encouraging for future exploration in research. In addition, we hypothesise that studies primarily focus on comparing devices to performance on the ALS-FRS(R) questionnaire. We hypothesise that pwMND will find these devices to be acceptable to use.

## Methods

### Search strategy

We completed a systematic and unbiased literature search on the 13th June 2022, adhering to PRISMA guidelines for conducting and reporting systematic reviews, attached in Appendix 1_PRISMA Guidelines. We searched EMBASE with the terms “amyotrophic lateral sclerosis” OR “motor neuron(e) disease” AND “devices” with the headings expanded to include all relevant sub-headings for devices. We also searched PubMed using (amyotrophic lateral sclerosis [MeSH Terms]) OR (motor neuron disease [MeSH Terms]) AND (devices [MeSH Terms]). In addition, we searched Google Scholar with the search terms “amyotrophic lateral sclerosis” OR “motor neurone disease” AND “wearable devices”, as “devices” alone provided an unmanageable number of results on this database.

Outside of the United Kingdom MND is primarily referred to by its most common subtype, amyotrophic lateral sclerosis (ALS), as a result both terms were included. No language or date restrictions were applied. Reference lists of returned search results were also screened for additional suitable articles.

### Screening for eligibility

**Fig. 1:**
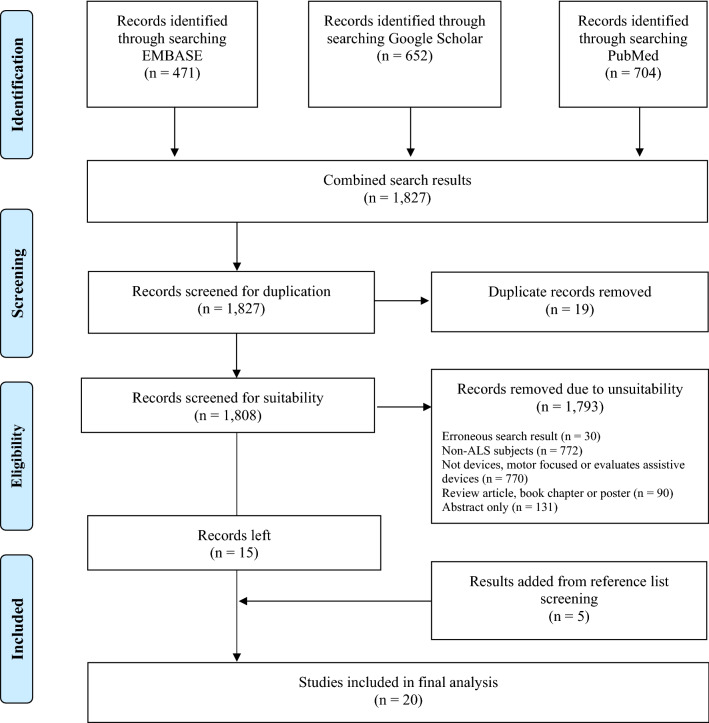
From Moher et al. [18]. For more information, visit www.prisma-statement.org

Results were screened for suitability by two independent reviewers, with any disputes resolved by a third reviewer. Full details of inclusion and exclusion criteria used are provided in Table 1 and the number of results included at each stage in the screening process is outlined in Fig. 1.

**Table 1:** Inclusion and exclusion criteria

Inclusion criteria	Exclusion criteria
• Cohort study, case–control study, feasibility study, letter, case series or case report AND; • Study population includes people with motor neuron disease o(including any of the listed subtypes: amyotrophic lateral sclerosis, progressive muscular atrophy, primary lateral sclerosis or progressive bulbar palsy) AND; • The device measures an aspect of motor system pathophysiology (such as movement, strength or impedance) OR; • The device output is used to assess progression of physical symptoms OR; • Gait analysis when focussed on progression or evaluation of declining function	• Not including any participants with any form of motor neuron disease • Paediatric or non-human study population • Review articles, conference abstracts, book chapter, poster or clinical trial • Electronic medical device is invasive or implanted • Device measures speech, respiratory function, energy expenditure, cognitive function or an aspect of disease unrelated to motor pathophysiology • Sensor output used for rehabilitative or assistive purposes (e.g. user–computer interface, communication aid and prosthetic) • Gait analysis focussed on identifying pathological gait, or differential diagnosis between neurological conditions

### Data extraction

Two reviewers extracted information from each search result on the devices used and study participant characteristics. We also noted any additional assessments used to evaluate the devices’ suitability and evaluation of participant feedback on the suitability of these devices.

### Risk of bias

Two authors (EB, TF) independently assessed the methodological quality of all included studies using the Quality Assessment of Diagnostic Accuracy Studies (QUADAS-2) tool for quality assessment questionnaire [19]. Studies were judged to either have high or low risk of bias for each domain based on composite assessment of 13 questions. All studies fitting inclusion criteria were included in the review despite their risk for bias.

## Results

An overview of the studies including the specific devices and comparative assessment tools used for concurrent validity is summarised in Table 2 with the full dataset available in Appendix 2_Project Data.

**Table 2:** Overview of included studies

Lead author	Total number of participants	Number of participants with MND	Information on disease subtype	Length of follow-up	Frequency of data collection	Function assessed	Motor sensor	Additional assessments	Comparison devices	Data on participant experience reported?	Company providing device	Cost of device
Andres (2017) [16]	100	100	No subtype data	15 months	Every 5 months	• Upper and lower limb strength	• Accurate Test of Limb Isometric Strength (ATLIS)	• ALS-FRS(R)	None	No	ATLIS	No data
Bakers (2021) [21]	88	45	91% ALS 7% PMA 2% PLS	Single time-point	Not applicable	• Upper and lower limb strength	• Portable fixed dynamometer	• ALS-FRS(R)	Hand held dynamometer	No	Not applicable	No data
Berry (2019) [13]	23	22	No subtype data	6 months	Every 3 months	• Speech • Cognition • General functioning (self-reported ALS-FRS(R), communication and movement logs)	• Smartphone (Beiwe app)	• ALS-FRS(R) • ALS-CBS	Vital Capacity (Easy One portable digital spirometer)	No	Onnela Lab at Harvard University	No data
Beukenhorst (2021) [22]	8	8	No subtype data	3 months	2-month-long time periods	• General activity calculated from location data	• Smartphone (Beiwe app)	• ALS-FRS(R)	None	No	Onnela Lab at Harvard University	No data
Die Bie (2017) [23]	10	10	No subtype data	12 months	Approximately every 3 months	• Upper limb function	• Reachable workspace (Microsoft Kinect)	• ALS-FRS(R)	Forced vital capacity (Puritan Bennett Renaissance II Spirometer)	No	Microsoft	$41
Esser (2011) [37]	72	7	No subtype data	Single time-point	Not applicable	• Lower limb	• Inertial measurement unit	• Gait • Walking speed and distance	None	No	MTx Xsens	No data
Garcia-Gancedo (2021) [24]	25	25	No subtype data	12 months	Every 3 months Wear sensor 3 days a month	• Upper limb function • Speech	• Accelerometer (MegaFaros)	• ALS-FRS(R) • Cognition and fine motor skills (9-hole peg test)	• Heart rate variability (Mega Fast Fix electrode patch) Digital speech capture (microphone and computer)	Yes (reported adverse events, impact on activity and sleep quality)	Mega Electronics	$595
Geronimo (2021) [25]	30	30	84% ALS 14% PLS 2% PMA	Single time-point	Not applicable	• Lower limb	• Accelerometers	• Gait • ALS-FRS(R)	• None	No	MetaMotionR	$78
Godkin (2021) [36]	39	5	No subtype data	7 days	Daily	• Upper and lower limb function • ECG	• Accelerometer	• Temperature • Sleep • Mood • Cognition • Rankin scale	• None	Yes (De-brief interviews to discuss experience)	ActivInsights GENEActiv Originals Bittium Faros	$226
Kelly (2020) [26]	25	25	No subtype data	12 months	Wear device 3 days a month	• Activity • Heart rate variability • Speech	• Accelerometer (Mega Faros 180)	• ALS-FRS(R)	• Speech (digital data capture) • Heart Rate variability (Mega Faros 180)	No	Mega Electronics	$596
Londral (2013) [27]	6	3	No subtype data	6 months	Every 3 months	• Upper limb function (typing ability)	• Accelerometer (BioSignals PLUX)	None	None	No	BioSignals Plux	$95
Londral (2016) [38]	45	19	No subtype data	2–20 months	Every 4–6 months depending on progression	• Upper limb function (fine motor typing)	• Accelerometer	ALS-FRS(R)	None	No	No data	No data
Montero-Odasso (2017) [28]	500	40	No subtype data	36 months	Annually	• Lower limb function • Balance • Dual tasking (motor and cognitive tasks)	• Gait (GaitRITE® mat or PK Mas electronic walkway) • Accelerometer (GENEActiv)	• Neuro and ocular imaging • Neuropsychology (attention, executive, memory, speech production, language and visuospatial function) • Genomics	• None	No	GAIT Rite	$28,500
Oskarsson (2016) [29]	27	10	No subtype data	Single time-point	Not applicable	• Upper limb function	• Reachable workspace (Microsoft Kinect)	• ALS-FRS(R)	None	No	Microsoft	$41
Rutkove (2019) [30]	141	111	No subtype data	9 months	Daily data collection for 90 days, bi-weekly for 180 days ALS-FRS(R) weekly	• Activity	• Activity and sleep tracker (Mi Band) • Smartphone app (ALS AT HOME)	• ALS-FRS(R)	• Spirometer (AirSmart) • Electrical impedance myography (Skulpt Scanner) Speech (ALS AT HOME)	Yes (REDCap survey on patient-reported experience)	Xiaomi	$29
Rutkove (2020) [12]	113	113 61 in analysis	No subtype data	9 months	Daily for 3 months, twice weekly for 6 months ALS-FRS(R) weekly	• Activity • Upper limb • Respiratory function • Speech	• Activity tracker (Mi Band)	• ALS-FRS(R) • Patient-reported outcome measures	• Hand grip (Camry Handgrip Dynamometer) • Slow-vital capacity (Air Smart) • Electrical impedance myography (Skulpt Scanner) Speech (ALS at Home app)	Yes (participant-reported survey on experience)	Xiaomi	$29
Schefner (2018) [31]	106	46	No subtype data	8 months	Every 2 months	• Detect changes in muscle structure	• Myolex mView	• None	• NDD EasyOneVR Plus Diagnostic Spirometer MicroFet2VR hand-held dynamometer	No	Myolex	No data
Trevizan (2018) [32]	60	30	No subtype data	Single time-point	Not applicable	• Evaluate finger motion	• Microsoft Kinect • Leap Motion Control • Touchscreen laptop	• ALS-FRS(R)	• None	No	Microsoft Ultraleap	$41 $247
Van Eijk (2019) [10]	42	42	93% ALS 7% PMA	12 months	Wear sensor 7 days every 2–3 months Questionnaires daily	• Activity level (time active, metabolic equivalent, vector magnitude and movement)	• Accelerometer (ActiGraph GT9X)	• ALS-FRS(R) • HADS (Hospital Anxiety and Depression Scale) • Weight	• None	Yes (participants rate burden on ten-point scale)	Actigraph	$238
Vieira (2022) [33]	584	584	No subtype data	Single time-point	Not applicable	• Speech • Accelerometer	• Actigraph GT3X devices	• ALS-FRS(R)	• None	No	Actigraph	$238

1827 search results were identified of which 20 were studies eligible for inclusion (see PRISMA diagram in Fig. 1 for more details).

These 20 studies included 2044 participants (a mean of 102, range 6–584), 1275 (62%) of whom had MND. The remaining individuals were included as healthy controls or as people with other neurological conditions to offer comparison groups. 17 studies (85%) recorded participants’ ALS-FRS(R) scores [20], to evaluate concurrent validity of the devices [12, 13, 16, 21–33].

Length of follow-up and number of assessment points varied greatly across the studies, from a single time-point of assessment [25] to study duration of 36 months [28]. The median length of follow-up was 6 months, with a mean of 8 months. The number of assessment points, and different tools used, are summarised in Table 2. Devices were used as a part of larger study protocols, such as the Ontario Neurodegenerative Research Initiative (ONDRI), to differentiate presentation and progression across different neurological conditions [28, 34, 35].

Two studies reported on the feasibility of a fully remote research delivery model [12, 30]. Recruitment and informed consent were successfully completed using the internet and electronic transfer of medical records to confirm eligibility. Remote data collection was a more complex issue. Some participants struggled to set up study platforms independently, with up to 28% of participants with MND unable to record a first measurement [30] and only 15% of participants retained to the 9-months time-point (Table 2).

**Table 3:** Types of devices used

Type of device	Areas of functioning evaluated	Brand examples in included studies	Studies using
Activity monitor	• Heart rate • Personal activity • Breathing function • Stress • Sleep • Step count	Mega Fast Fix heartbeat sensor Mi Band Mega Faros Sensor	[24] [12, 30] [26]
Accelerometer	• Activity periods • Wear time • Metabolic rate • Energy expenditure • Steps taken	Actigraph BioSignals Plux Mega Faros 180 MetaMotionR MTXsens GENEActiv Originals Bittium Faros	[10, 33] [27] [26] [25] [37] [28, 36] [36]
Smartphone app	• Behavioural patterns • Sleep data • Social interactions • Physical mobility • Gross motor activity • Cognitive functioning • Speech production	Beiwe ALS AT HOME	[13, 22] [12, 30]
Gait	• Functional walking • Temporal and spatial parameters of movement	MetaMotionR GAIT Rite MTXsens	[25] [28] [37]
Movement sensor	• Reachable workspace for upper limbs • Fine motor skill on touch screen devices	Microsoft Kinect Leap Motion Control	[23, 29, 32] [32]
Spirometer	• Vital capacity	EasyOne AirSmart Puritan Bennett Renaissance II	[13] [12, 30] [23]
Electrical impedance myography	• Biomarker of neuromuscular health	Skulpt Scanner Myolex mView	[12, 30] [31]
Computerised microphone	• Speech capture	Not specified	[24, 26, 33]
Dynamometry	• Limb and grip strength	Accurate test of limb isometric strength (ATLIS) Portable fixed dynamometer (PFD) Camry handgrip dynamometer	[16, 21] [12, 30]

Devices may have multiple functionalities and not all functionalities will be automatically enabled

Five of the studies explored a form of participant experience on the suitability of the devices to people with MND. Participants completed questionnaires, reported adverse effects and rated the burden of using devices. In one study, semi-structured interviews were undertaken to provide participants with the opportunity to discuss their experiences [36]. Concerns ranged from the fairly innocuous, limited clothing options and worry of losing regarding the ActiGraph [10], to more serious adverse effects of skin and subcutaneous tissue disorders in 6 of 25 participants, resulting in 2 participants withdrawing from the study [26].

### Devices used

Accelerometers were used in nine studies [10, 25–28, 33, 36–38] including those using an accelerometer and additional sensors (gyroscope and magnetometer) in an inertial measurement unit. These sensors were used to evaluate spatio-temporal parameters of gait specifically in three studies [25, 28, 37]. Five studies evaluated lower limb function using accelerometers worn on the waist or ankle during walking tasks [25, 28, 33, 36, 37]; two of which also involved wearing one the wrist to assess upper limb functioning [33, 36]. In addition, two studies used small accelerometers on participants’ fingers to evaluate fine motor skills through measuring typing speed, strength and accuracy [27, 38].

The Microsoft Kinect sensor uses a single camera system to evaluate depth and an individual’s motion, that can be used to capture reachable workspace [39], a clinically relevant measure of upper limb function, providing information on capacity to move the arms and reach within their environment. The Microsoft Kinect sensors were used to evaluate upper limb function, through mapping reachable workspace, in three studies [23, 29, 32]. One study explored the suitability of non-immersive virtual reality tasks as a method of assessing upper limb functionality and cognition, comparing touchscreen laptops, Microsoft Kinect motion sensor and finger motion sensor system Leap Motion Control^®^ [32].

Smartphone applications, such as the Beiwe [13, 22] and ALS AT HOME apps were used [12], offering frequent remote data collection, often using a wide range of endpoints. Smartphone data were categorised as active (directly inputted by participants) and passive (using existing smartphone data such as GPS and call logs), used to calculate activity [13, 22]. Participant-completed ALS-FRS was supplemented with additional questionnaires, motor tests, digital spirometry and cognitive testing.

Six studies evaluated ‘activity monitors’ that collect, and present, data on measurements such as time spent active, sleeping and metabolic equivalent task (MET). MET is a calculation based on body weight to estimate the number of calories burned during an activity or ‘task’ [40]. Monitoring of general activity using wearable devices in studies also enabled researchers to collect data outside of the artificial clinic environment of changes in the participants’ daily functioning, with minimal burden on participants. The Mi Band [12], ActiGraph [10] and Mega Faros sensor [26] were used to evaluate real-world activity levels and heart rate variability during rest and exercise. Activity monitors were combined with additional devices to evaluate respiratory function, such as the AirSmart spirometer [30], and upper limb function, using the Camry digital handgrip dynamometer [12].

Some studies focussed on devices that specifically aimed to evaluate progression in muscle strength. Electrical impedance myography, which works through the application of low-intensity electrical currents to the muscles, was shown to be potentially suitable to evaluate MND progression [31]. Two studies explored the use of fixed and portable dynamometry devices to assess global and precise muscle strength [16, 21], with hand-held dynamometry devices a common comparison measure to establish the suitability of new exploratory devices [12, 31].

### Data analysis

Accelerometer devices collect raw data in three axes from the primary accelerometer sensor. The raw data are analysed using either data visualisation tools and pre-existing algorithms provided by the vendors or devising new data analysis models [26]. Working with the raw data to generate novel scoring thresholds enables investigators to replicate findings in the future studies, compare participants and provide preliminary validity data on the prognostic probability of the devices used.

The data from activity monitors and accelerometers are used to correlate the level of change expected based on standardised tests of disease progression and functionality; in MND studies, this is primarily the ALS-FRS(R). Other studies use raw data from the devices to quantify the individual’s movements when performing a standardised clinical measurement or motor function assessments such as the 6-minute walking test [28], arm raising [29] or typing [27]. Assessments that may be clinically relevant to people with MND may not yet have reliability data on which devices are suitable to evaluate motor functionality during them, a key foundation in considering the suitability of a device to this population.

### Risk of bias in included studies

Risk of bias in the included studies is shown in Appendix 3_QUADRAS Data. No study fulfilled all QUADAS-2 criteria for low risk of bias. 31% (126/408) responses were deemed “Unclear” as the information regarding sample decisions, reference and index tests used were not available in the study report meaning the conclusions regarding risk of bias were affected by lack of data availability. However, for all the studies, there was low concern that the index test used, the interpretation of the index test or the participants included, differed from the review question.

## Discussion

Digital devices have provided an alternative to traditional questionnaire-based assessment methods to detect progression in clinical trials [41] and research studies [42] in a range of neurological disorders. These devices offer a potential new direction in MND research and clinical care and this review explores the current landscape in this area; the device types used and the suitability and acceptability for pwMND.

### Establishing suitability of devices

Studies in this review reported that device outcome measures correlated with the ALS-FRS(R), suggesting that devices have concurrent validity with traditional measures of disease progression [12, 13, 16, 21–33].

Accelerometer endpoints (average daytime active, percentage of daytime active, total daytime activity score and total 24-h activity score) showed moderate to strong correlations with ALS-FRS(R) scores over a period of 48 weeks [26]. Strong associations between accelerometer endpoints and ALS-FRS(R) scores for up to 21 months and accelerometer data indicated less variability over time [10].

Worsening total ALS-FRS(R) scores, and declining ALS-FRS(R) upper limb sub-scores, were associated with reduced reachable workspace evaluated through Kinect sensors [29]. De Bie et al. [23] also demonstrated the potential utility of device outcome measures, as in their study Kinect sensors were able to detect change in upper limb function when the ALS-FRS(R) did not indicate any significant change over a 1-year period. Scores from activity monitors, specifically the amount of time spent active, correlated with global and motor-specific scores on the ALS-FRS(R), suggesting the potential utility of these devices to evaluate function with low burden to participants [25].

Devices also offered the opportunity to take ALS-FRS(R) data collection out of the clinic, with self-reported ALS-FRS(R) scores correlating highly with clinic-based assessments [13], working towards establishing app-based evaluation and fully remote studies [30] as a potentially suitable alternative to burdensome clinic appointments for people with MND involved in research.

However, the sensitivity of the ALS-FRS(R) to detect smaller changes in functioning is limited [5]. When establishing the suitability of devices for people with MND, concordant validity with existing measures is a helpful starting point, but should not be the only consideration for a devices’ utility in research and clinical evaluation. Responsiveness to change in other physical markers of progression, such as respiratory function and muscle strength, may also be relevant.

### Responsiveness to change over time

Only three studies explored the devices at a single time-point [25, 29, 37]. The remaining studies (*n* = 17) explored change in motor symptoms over time, between 2-week and 36-month study duration.

As when evaluating any progressive disorder, establishing the suitability of these devices to detect change was crucial. Devices were often compared to established measures of disease progression, to establish the ability to detect expected decline and ultimately detect potential treatment effects as biomarkers in clinical trials [31].

The broad range of aspects of functioning which can be evaluated using devices is particularly useful in a condition such as MND with heterogeneous presentation and progression. Devices offer objective measures of both global and precise decline and have been shown by studies in this review to successfully differentiate disease progression [10] and discriminate between neurological conditions [37].

Increasing the length of follow-up, and the number of measurements, to evaluate the reliability of a device across repeated measurements will be a key avenue for future work.

### Establishing acceptability of devices

Current data on the acceptability of devices to people with MND were limited, with only five (36%) studies reporting data on participant experience [10, 12, 24, 30, 36]. For the studies that did report on participant experience, feedback was generally positive with participants reporting low burden, an improved sense of control over their condition and minimal impact on their day-to-day activities [10, 12].

The logistical challenge of remote data collection, potential risk to participant safety, and shift in onus on the participant to collect the data, must be carefully managed by clinical and trial teams. Feedback from participants, rates of adverse events and attrition must be closely explored when evaluating the suitability of a potential device for use in this population.

### Remote data collection

A key benefit of wearable devices and other health technologies to evaluate disease progression in MND is their portability and suitability for remote data collection. A recruitment, consent and data collection process that was either fully or partially remote would significantly reduce the burden placed on individuals who wish to participate in clinical trials. It would also minimise burden of repeated travel in this vulnerable group, thereby minimising attrition.

Remote data collection using devices presents unique challenges associated with managing and reducing missing data. Studies involving remote data collection reported missing data due to insufficient device charge and participant adherence to protocol of wearing devices. Inability to use the devices and begin data collection may also be problematic, with one study reporting 13% of healthy controls and 28% of pwMND were unable to obtain a first measurement using the technology provided. Only 15% of participants were retained to the 9-month time-point. With only 15% of participants remaining at the 9-month time-point, our ability to draw conclusions from remotely collected data in these studies is limited [12].

### Participant-led data collection

The shift to participant-led data collection may be contingent on individuals having a degree of technical knowledge and confidence in their ability to use devices, potentially affecting those who opt to engage [11, 12]. This may bias samples in studies using health technology, for example towards younger individuals, with greater digital literacy and those less affected by upper limb weakness. Clinicians and researchers may pre-emptively address this concern by reducing skills required for people to participate, providing adequate instructions for use, and ongoing technical support. The use of cloud-based data collection methods may also reduce the onus on participants, enabling remote monitoring of adherence and data management from devices.

The inclusion of devices in study design may result in greater participant burden ranging from the intrusion of remembering to wear sensors to attending more remote appointments. It is important that these factors are considered in study design. This burden may also be felt by caregiving relatives and friends as people with MND experiencing motor decline or cognitive impairment may require prompting and physical assistance from a caregiver to adhere to study requirements.

### Choice of device

The aim of this study was to systematically review the current landscape of research using devices to explore motor progression in MND. Device suitability is dependent upon the research aims, the intended participant group and project resources but more research into the properties of these devices, and their acceptability to people with MND can help inform future decisions.

A key aspect requiring consideration is the current lack of consensus regarding choices of device for digital data capture. This lack of consensus, linked to the small sample sizes [27] and low quality of existing evidence [29, 37], introduces uncertainty, and may limit uptake in clinical trial design and also patient care.

Prospective studies, with larger sample sizes, longer follow-up durations and direct feedback from device users [43] are required to evaluate the utility of devices and establish which devices are most suitable in MND. The development of strategic guidelines would be beneficial to harmonise approaches and inform future study design and clinical care integration. Many devices contain multiple sensors and investigators must balance the benefits of additional data collection with concerns over decrease battery life and greater data storage requirements. If using accompanying software, the type available for data analysis and data visualisation is relevant in informing design decisions.

### Data analysis and management

A further potential challenge for researchers incorporating devices in trial design, and clinicians integrating devices into care, is how to use device data to evaluate progression. A clear, pre-defined plan for evaluating digitally derived data, correlating with existing validated outcomes and determining thresholds of progression are crucial [44].

As with any outcome measures, the findings from studies using devices can be affected by missing data. Data points may be compromised due to technical issues of erroneous recordings, transferring and storage of data. Studies included in this review reported issues with missing data affecting the ability to draw clinical conclusions from results, due to participants withdrawing early due to adverse effects [24] or being unable to use the technology to collect data [30]. Investigators must manage the risk of missing data, and develop study-specific plans to account for this in data analysis for future studies.

If investigators intend to use raw data for analysis, how the data is stored and organised and the availability of groups with expertise in this type of data analysis must be a crucial consideration. Concerns regarding data security and adherence to privacy regulations may also be a barrier to integrating technologies in study design. Providing detailed data management plans, reviewed by specialists in data security will help to pre-emptively address concerns of prospective participants, regulatory bodies and funders.

### Cost of devices

A potential concern for prospective users, regulatory bodies, funders, clinicians and research teams is the cost of providing technologies. Accessibility of these devices may be limited by the cost of providing equipment such as tablets and computers, or purchasing specialist voice recognition or eye gaze software. Approximate costs for devices, where available, are reported in Table 2. Resource is also required for storage and interpretation of data requiring interdisciplinary collaboration with clinicians, data scientists, information governance, and IT security. Ensuring future work is not limited in scope by access to devices, will be difficult to address without additional funding for MND care and research.

### Conclusion

Overall, the use of devices for measuring disease progression in MND is a promising direction of research. The reviewed literature was primarily proof-of-concept, exploratory studies with shorter periods of follow-up and smaller sample sizes, limiting the conclusions that can be drawn from the findings. In addition, a large amount of data were unavailable to determine risk of bias accurately, and for the information available, a high risk of bias was indicated. The COVID-19 pandemic has highlighted the importance of implementing remote assessment, using the types of technology discussed in this study, for people with MND [45]. Devices offer the opportunity to decentralise trial delivery and reduce the burden felt by participants previously required to travel to additional appointments.

### Future work

Clinicians and trialists designing research to incorporate these devices will face a unique set of considerations and challenges. The body region to be assessed, ease of use, frequency and sensitivity of sampling, reliability, cost to purchase, battery life and storage capacity of devices must be evaluated. The shift towards telemedicine in clinical care may offer valuable insights into delivering effecting remote research opportunities for people with MND [46].

This study indicates a variety of devices are potentially suitable to measure physical symptoms in MND and potentially useful as additional outcome measures in trials. The multi-systemic impact of MND presents multiple potential targets for evaluation as potential technological biomarkers, including speech, motor function, cognition and overall functional ability [47].

As there are a number of potentially suitable devices, the decision on what to use must also consider acceptability to participants, cost, area of function to assess and sensitivity to change. Identifying relevant devices, establishing their suitability and providing clear procedures for integration into health research, specifically for MND, are outlined by Van Eijk et al., who highlight the importance of digital biomarkers [44].

When establishing the responsiveness of a device, future research should evaluate progression of each currently affected area of the body, not just as covariance with total scores from existing measures of progression such as the ALS-FRS(R), which may not be sensitive to detect smaller changes in function. Future work should focus on developing guidelines for clinicians and researchers on available devices, suitability for MND and aspects of functioning measured.

## Supplementary Information

Below is the link to the electronic supplementary material.

Supplementary file1 (DOCX 37 KB)

Supplementary file2 (XLSX 22 KB)

Supplementary file3 (XLSX 30 KB)

## Data Availability

Appendix 2_Project Data.
